# Genetic Profiling Reveals High Allelic Diversity, Heterozygosity and Antigenic Diversity in the Clinical Isolates of the *Theileria annulata* From India

**DOI:** 10.3389/fphys.2019.00673

**Published:** 2019-06-07

**Authors:** Sonti Roy, Vasundhra Bhandari, Debabrata Dandasena, Shweta Murthy, Paresh Sharma

**Affiliations:** ^1^National Institute of Animal Biotechnology, Hyderabad, India; ^2^Manipal Academy of Higher Education, Manipal, India

**Keywords:** *Theileria annulata*, genetic profiling, microsatellite markers, genotyping based sequencing, antigenic diversity

## Abstract

Tropical theileriosis caused by *Theileria annulata* infection is a significant livestock disease affecting cattle health and productivity resulting in substantial monetary losses in several countries. Despite the use of an effective vaccine for disease control still, a high incidence of infection is reported from India. One of the many reasons behind the ineffective disease control can be the existence of genetically diverse *T. annulata* parasite population in India. Therefore, studies focusing on understanding the genotypes are warranted. In this study, we have performed a genetic analysis of the Indian *T. annulata* field cell lines and the vaccine line using microsatellite markers, Genotyping based sequencing (GBS) and *tams1* gene polymorphism. The degree of allelic diversity and multiplicity of the infection was determined to be high in the Indian population. No geographical sub-structuring and linkage disequilibrium were observed in the population. High population diversity was found which were similar with countries like Oman, Tunisia, and Turkey in contrast to Portugal and China. The presence of multiple genotypes as determined by microsatellite marker genotyping, GBS analysis and *tams1* gene polymorphism point toward a panmictic parasite population in India. These findings are the first report from India which would help in understanding the evolution and diversity of the *T. annulata* population in the country and can help in designing more effective strategies for controlling the disease.

## Introduction

In India, tropical theileriosis accounts for high mortality and economic loss to the livestock industry (George et al., 2015). The disease is caused by *Theileria annulata* belonging to the phylum apicomplexan, its life cycles requires a vector (ixodid tick) and host (cattle or buffalo). Currently, a live attenuated schizont lymphocyte cell line is used as a vaccine for controlling the *T. annulata* infection in India (Chakraborty et al., 2017). Despite the availability of a licensed vaccine and a theileriocidal drug, buparvaquone (BPQ), the disease still represents a severe threat to animalhealth and productivity especially to the crossbreed cattle. The major disadvantages with the current vaccine are the requirement of re-vaccination after every 3 years, maintenance of cold chain and transportation in liquid nitrogen. Additionally, cases of drug resistance were also reported from the field (Mhadhbi et al., 2010, 2015; Sharifiyazdi et al., 2012). Another important aspect which may reduce the vaccine effectiveness will be the presence of heterogeneous parasite population in the field. Hence, it is essential to investigate the genetic diversity of the *T. annulata* parasites currently prevalent in India.

The life cycle of the *Theileria* parasites involved both asexual and sexual reproduction, with later responsible for genetic recombination but restricted to the tick (Mans et al., 2015). As parasite diversity is mainly driven by recombination events, it is having a crucial role in deciding the fate of the parasite control measures. Previous reports have shown genetic diversity among the *T. annulata* parasites using a panel of 10 micro and minisatellite markers, *tams1* gene PCR, and indirect fluorescence assay (IFA) (Shiels et al., 1986; Dickson and Shiels, 1993; Katzer et al., 1998; Gubbels et al., 2000a,b; Wang et al., 2014). The genetic diversity of *T. annulata* parasites using microsatellite marker have been reported previously from various countries like Oman (Al-Hamidhi et al., 2015), Sudan, Tunisia, Turkey, (Weir et al., 2007, 2011) Portugal (Gomes et al., 2016), and China (Yin et al., 2018). However, the parasite diversity studies from India are very scarce; the few available reports are based on IFA and PCR using 18S *rRNA* and *tams1* genes (Manuja et al., 2006; Wang et al., 2014; George et al., 2015). With significant advancement in the field of genomics and genotyping and with the availability of the *T. annulata* whole genome sequence, the likelihoods of genotype-based population genetic studies are open. The genetic resolution of the parasite diversity can be improved by studying more number of markers, like in closely related parasite *Theileria parva and Plasmodium falciparum* (Campino et al., 2011; Henson et al., 2012; Hayashida et al., 2013; Hemmink et al., 2016; Chen et al., 2017). Genotyping based sequencing (GBS) is one such next-generation sequencing based strategy which can identify variants/SNPs for high diversity species with accuracy and cost-effectiveness (Elshire et al., 2011; Gurgul et al., 2018).

It is significant to study the genetic diversity of the *T. annulata* parasite for developing new vaccines, new therapies, and designing more effective disease controlling strategies. In this study, we have used the *T. annulata* live attenuated vaccine line and parasite cell lines isolated from animals infected by *T. annulata* for understanding the antigenic, allelic, and genotypic diversity of the Indian parasite population. Our study will not only help in understanding the genetic diversity in the Indian *T. annulata* parasites but also provide a comparative account with parasites from different countries and vaccine line currently in use.

## Materials and Methods

### *In vitro* Culture of *Theileria* Cell Lines

*Theileria annulata* infected lymphocyte cell lines were established by culturing the peripheral blood mononuclear cells (PBMCs) isolated from the whole blood of the infected cattle from the states of Telangana and Andhra Pradesh in India. The infected animals were cross breed (Jersey breed) and had never been vaccinated against tropical theileriosis. The cell lines were grown in the RPMI 1640 medium at 37°C as per standard protocol (George et al., 2015). The vaccine cell line (V) was purchased from the Indian Immunologicals Ltd., Hyderabad. The DNA was extracted from the parasite lines using standard phenol/chloroform method and was confirmed by PCR using *T. annulata* surface protein (*Tasp*) primers as mentioned in **Table 1**.

**Table 1:** Primers used in the study.

Primer name	Sequence (5′–3′)	Product size	TM
Tasp For	ATGAAATTCTTCTACCTTTTTG	1065 bp	60
Tasp Rev	ACAACAATCTTCGTTAATGCGA		
Tams For	ATGTTGTCCAGGACCACCCTC	858 bp	55
Tams Rev	TTAAAGGAAGTAAAGGACTGA		


### Micro and Mini Satellite Marker Analysis

The known micro (TS 5, 9, 12, and 16) and minisatellite (TS 6, 8, 15, 20, 25, and 31) markers were used for genotyping *T. annulata* parasites (Weir et al., 2007). The 5′ end of the forward primers of all ten loci were fluorescently labeled with FAM dye. The PCR was done using locus-specific primers to amplify all the markers as mentioned before (Weir et al., 2007, 2011). The purified PCR product was then run on the ABI 3730Xl genetic analyzer with ROX labeled GS500 standard size marker for genotyping analyses. Further analyses was carried out by GeneMarker V2.7. The allele count was analyzed for each locus in the range of 100–500 bp. The peaks higher than 32% of the predominant peak was used for scoring allele to avoid minor amplification or PCR artifacts and a predominant allele at each locus is used to prepare the multilocus genotype (MLG) for each sample (**Table 2**; Weir et al., 2007, 2011). The MLG data was used to calculate the genetic distance matrix and heterozygosity at each locus using GenAlEx 6.5 software (Peakall and Smouse, 2012). The genetic distance matrix was used for performing PCoA analysis using GenAlEx 6.5 software. ^A^I_S_ (index of Association), V_D_ (Variance of the pairwise differences), L_MC_ (Upper 95% confidence limits of Monte Carlo simulation), and L_PARA_ (parametric tests) were calculated using Lian 3.7 (Haubold and Hudson, 2000).

**Table 2:** Allele diversity among the different parasite strains for the 10 micro and Mini satellite markers.

Sample ID	Chr 1		Chr 2		Chr 3			Chr 4		
	TS15	TS16	TS20	TS31	TS8	TS9	TS12	TS5	TS6	TS25
C1	3	4	3	4	3	2	4	3	6	1
C2	2	1	5	12	6	3	3	2	7	6
C3	5	4	4	15	12	3	9	4	20	2
C4	7	7	3	7	4	2	1	2	20	2
C5	7	2	6	6	4	7	11	5	6	5
C6	5	1	4	7	12	2	2	1	4	2
V	3	2	3	16	3	4	5	2	20	2
Tunisia	8	12	13	18	24	22	18	8	16	7
Turkey	7	7	10	10	8	9	10	6	10	7

*Chr, Chromosome*.

### Genotyping Based Sequencing

The DNA isolated from the 6 parasite strains (C1, C2, V, C4, C5, and C6) was checked for its quality and quantity using Nanodrop and Qubit. Genomic DNA (300 ng) was digested by double digestion using Pstl-HF and MluCl restriction enzyme at 37°C for 4 h. The resulting digested fragments were directly ligated to a pair of restriction site-specific adapters at 20°C for 1 h using T4 DNA ligase. The adapter-ligated fragments were subjected to PCR amplification (10 cycles) to amplify the adapter-ligated fragments and to add sample specific dual index barcodes (Nextera XT v2 Index Kit, Illumina, United States). The amplified Illumina-compatible sequencing library was quantified by Qubit fluorometer (Thermo Fisher Scientific, MA, United States) and its fragment size distribution was analyzed on Agilent 2200 Tape Station. The library was used to run the Illumina single-end sequencing (75X1, NextSeq) for all the samples. The raw reads quality from the Illumina run were checked using FastQC. *T. annulata* genome specific reads were separated through alignment using Bowtie2, and aligned reads from each of the samples were used for variant identification. A merged FASTQ file was generated for all the six samples using the good reads for the organism. This merged FASTQ file was aligned to the reference genome of *T. annulata* (GCF_000003225.3) using Bowtie2. The alignment quality was checked using Qualimap software. To filter out low-quality SNPs/variants, the TASSEL-GBS pipeline was used for filtering minor allele frequency (MAF) and inbreeding coefficient. The consensus sequence generated from the run for each cell line was used for performing a multiple sequence alignment using ClustalW algorithm. The Ankara genome sequence was also included in the analysis as a control. Gblocks program was used to identify the conserved stretches in the genomes. Finally, MEGA7 program was used to calculate the phylogenetic distance between the parasite strains and for drawing a phylogenetic tree using the consensus sequence (Wang et al., 2014). The evolutionary history and phylogenetic tree were generated using the neighbor-joining method.

### *Tams1* Based Antigenic Diversity Analysis

The DNA isolated from the seven parasite strains was used for amplifying the *tams1* gene using for and rev primers (**Table 1**). The purified PCR products of 858 bp were then cloned into the pBSK vector using the TA cloning method followed by Sanger’s sequencing to confirm the *tams1* gene. The sequencing results were further confirmed by checking the specificity of the sequence using blast analysis. The previously published *tams1* gene sequences from the three continents Asia, Africa and Europe were used for calculating the phylogenetic relationship between the strains using MEGA7 software (Wang et al., 2014). Accession number of the sequences that were used U222888.1 (Ankara), Z48739.1 (Ankara), GU130193.1 (Iraq: Kurdistan), AF214819.1 (Mauritania), JX683683.1 (Gansu: China), AF214911.1 (Turkey: Ankara), AF214872.1 (Tunisia: Northern), AY672541.1 (Iran), EU563912.1 (China: Xinjiang), AF214828.1 (Portugal), AF214860.1 (Italy: Northern Sicily), AF214812.1 (Spain: Cáceres), EF618726.1 (India: Chennai), EF618728.1 (India: Chennai), JX648210.1 (South India: Tamil Nadu), AF214840.1 (India: Hissar), AF214891.1 (Tunisia: Northern), AF214861.1 (Italy: Northern Sicily), AF214908.1 (Turkey: Ankara), AF214806.1 (Spain: Cáceres), AF214797.1 (Bahrain), GU130190.1 (Iraq:Kurdistan region), AB690863.1 (Srilanka: Polonnaruwa), GU130192.1 (Iraq: Kurdistan region), EF092918.1 (Iran: Qazvin Boein Zahra). These sequences were collected from the NCBI site and imported in MEGA7 software and aligned using the ClustalW method. The evolutionary history and phylogenetic tree were generated using the neighbor-joining method.

### Ethics Statement

Collection of less than 5 ml of blood, in accordance with national legislation, is exempt from ethical approval requirements. Further, professional veterinarians collected blood samples after obtaining oral consent from farm owner.

## Results

### High Allelic Diversity Observed in the Indian Parasite Populations

Microsatellite analysis was done for the seven parasite cell lines (C1, C2, C3, C4, C5, C6, V) using the 10 markers. All the markers showed high polymorphisms in the Indian parasites although in few cell lines a single allele was observed for TS16, TS12, TS5, and TS25. The diversity of alleles ranged from one to twenty among the Indian parasite cell lines. In C3, C4, and V, highest polymorphism (*n* = 20) was observed for TS6 marker. Whereas low polymorphism (*n* = 1) was observed against markers TS16, TS12, TS5, and TS25 in C2, C6, C4, and C1 cell lines (**Table 2**).

**FIGURE 1:**
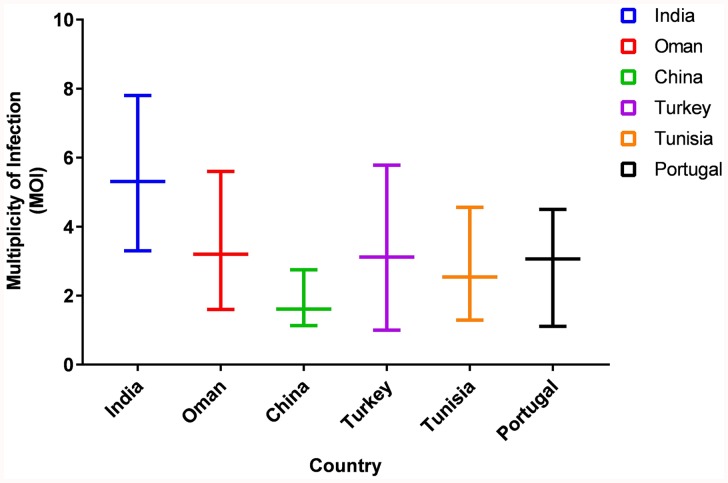
Comparison of Multiplicity of Infection (MOI) in Six countries. The graph represents the range of MOI in six countries.

The MOI for the Indian parasitic cell lines ranged from 3.3 to 7.8, with an average MOI of 5.31 (**Figure 1**). The MOI range of the Indian population was higher when compared to the parasite from Sudan, Oman, China, Portugal, and Turkey which ranged from 1.0 to 5.78.

### High Population Diversity, Linkage Equilibrium and No Geographical Sub Structuring Seen in the Indian Population

**FIGURE 2:**
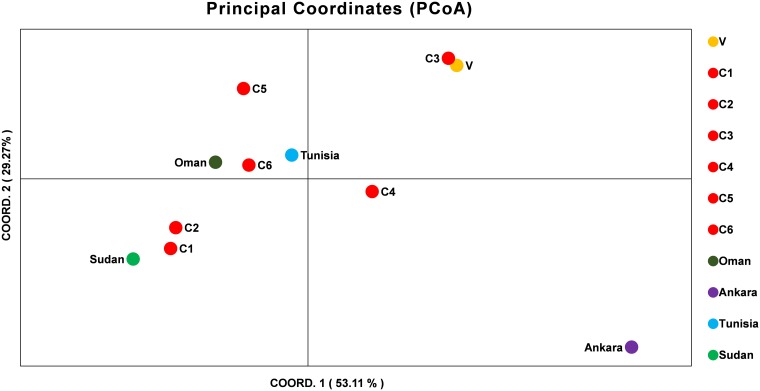
Principal Component Analysis (PCoA) of *T. annulata* parasite population from India, Oman, Ankara, Sudan, and Tunisia. PCoA analysis based on the MLG profiles of the *T. annulata* parasites was done to understand the geographical distribution of the parasites in different countries. The two coordinates along with the proportional of variance (%) used for doing the analysis are shown in *X* and *Y* axis.

The allelic profiles were used to create MLG data set for each marker using the predominant allele for the Indian population. The MLG data was further used for calculating heterozygosity, linkage disequilibrium and the genetic distance between the parasite strains. The estimated heterozygosity (H_e_) for the Indian population was found to be 0.82041 which was determined by using the predominant allele dataset for each marker and averaged across all ten loci (**Table 3**). For understanding the Indian population differentiation, linkage analysis was performed by calculating values of I^S^_A_ = 0.0191 and V_D_ = 0.4571 which was found to be less than L_MC_ = 0.6571 and L_PARA_ = 0.5732 indicating linkage equilibrium (LE). The PCoA analysis showed no specific geographical distribution between the Indian parasites when compared to the parasites from other countries like Oman, Sudan, Tunisia, and Turkey. The analysis showed C1 and C2 parasites cluster with Sudan while C5 and C6 with Tunisia and Oman suggesting allelic similarities. The C3 and V clustered together in a separate cluster while C4 and parasite from Turkey didn’t cluster with anyone. The variation among the samples was found to be 53.11 and 29.27% for the first and second coordinates of the graph, respectively (**Figure 2**).

**Table 3:** Comparison of heterozygosity and linkage equilibrium analysis.

Country	He	I_A_^S^	V_D_	L_MC_	L_PARA_	Linkage
India	0.82041	0.0191	0.4571	0.6571	0.5732	LE
Oman	0.8307	0.019	1.4595	1.29	1.29	LD
China	0.5	0.1224	2.6253	1.5727	1.5635	LD
Turkey	0.8817	0.0207	1.0671	0.9495	0.944	LD
Tunisia	0.8841	0.0117	0.9534	0.9128	0.9088	LD
Portugal	0.625	0.0272	2.0247	1.8679	1.8521	LD

*He, Estimated heterozygosity; I_A_^S^, Standard index of association; V_D_, Mismatch variance (linkage analysis); L_MC_, Upper 95% confidence limits of Monte Carlo simulation; L_PARA_, Parametric tests; LD, Linkage disequilibrium; LE, Linkage equilibrium.*

### GBS Identified Genetic Polymorphisms Among the Different *Theileria* Parasites

**FIGURE 3:**
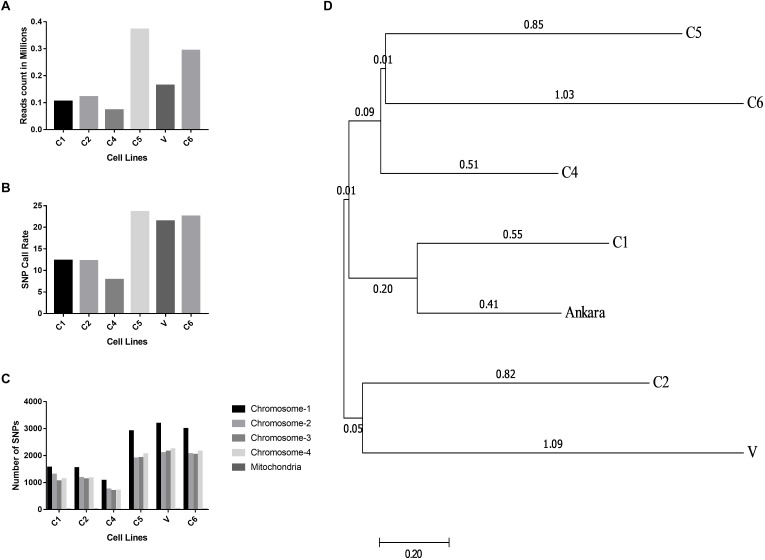
Distribution of reads, SNP call rates and Phylogenetic relationship determined from the GBS analysis. **(A)** The figure shows the read count in millions generated using the GBS experiments done for the six different *T. annulata* cell lines. **(B)** The figure shows the SNP call rate between the six cells lines used for the GBS when compared to the Ankara reference strain. **(C)** The figure shows SNP distribution within the four chromosomes and mitochondria for the six cell lines used for the GBS study. **(D)** The evolutionary distances were computed using the maximum composite likelihood method and are in the units of the number of base substitutions per site. The analysis involved seven nucleotide sequences. Bootstrap values are shown next to the nodes. Evolutionary analysis was performed with MEGA7.

The Illumina reads generated after sequencing were aligned with the *T. annulata* Ankara strain for identifying *T. annulata* specific sequences and SNPs in the Indian parasite cell lines. The alignment helped in constructing a consensus DNA sequence for the six parasites. The GBS sequencing results showed that all the six parasites were well represented and had an average read count of 0.18 million/sample (**Figure 3A**). The distribution of SNP call rate for the samples ranged from 7.87 to 23.61% and was represented for the individual samples (**Figure 3B**). The SNPs were well distributed among the four *T. annulata* chromosomes (Chr) with Chr 1 having the highest number 3210 SNPs followed by Chr 2, 3, 4, and mitochondrial DNA in all the samples (**Figure 3C**). The phylogenetic relationship between the Indian parasites (*n* = 6) were calculated based on the SNPs detected in the GBS sequences including the Ankara parasite sequence. The analysis identified C1 and C6 close to the Ankara sequence based on the genetic polymorphisms in the respective sequences. C4 and C5 were clustered together while C2 showed similarity with V (**Figure 3D**).

### Antigenic Diversity Was Observed for the *tams1* Gene Among the Parasites

**FIGURE 4:**
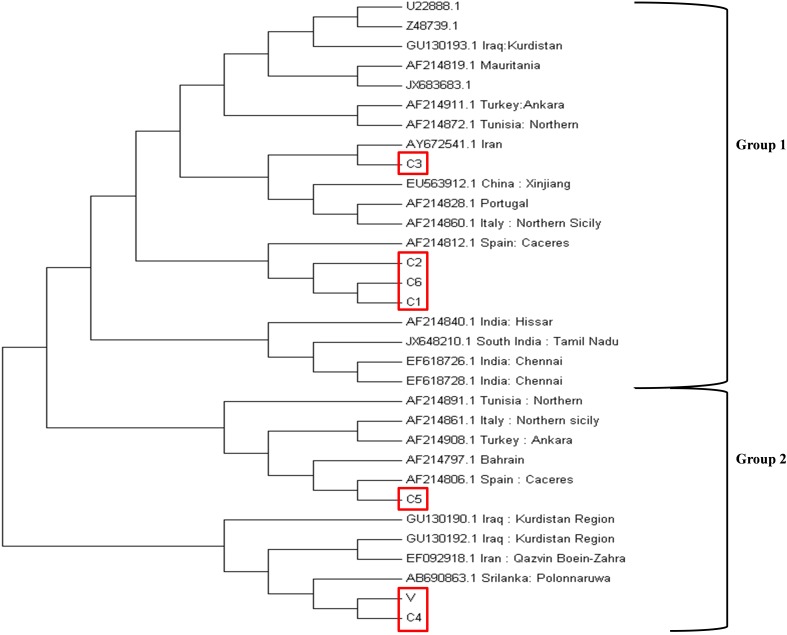
Phylogenetic tree based on the *tams1* gene sequences from different countries. The evolutionary history was inferred using the neighbor-joining method. The percentage of replicate trees in which the associated taxa clustered together in the bootstrap test (1000 replicates) are shown next to the branches. The evolutionary distances were computed using the maximum composite likelihood method and are in the units of the number of base substitutions per site. The analysis involved 32 nucleotide sequences. All positions containing gaps and missing data were eliminated. There were a total of 363 positions in the final dataset. Evolutionary analysis was concluded in MEGA7.

The phylogenetic analysis was performed based on the neighbor-joining method using the *tams1* gene sequences of seven Indian parasites (GenBank accession IDs MK034698-MK034704) and 25 previously reported parasites. The clusters were divided into two groups on the basis of previously published data (Wang et al., 2014). C1, C2, and C6 were clustered in group 1 while C4, C5, and V were in group 2 (**Figure 4**). The Indian isolates were distributed in both groups along with other countries like Spain, Italy, Tunisia, Iran, Bahrain, Turkey, and Iraq.

## Discussion

Despite the availability of a vaccine against *T. annulata*, theileriosis is frequently reported in India (George et al., 2015). The genetic diversity among the parasite strains can be one of the reasons for the failure to constraint the disease. This is the first report from India, where the genetic and allelic structure of the Indian parasites cell lines was investigated which revealed antigenic and allelic diversity among the parasite population. The parasite diversity data will be helpful in revamping or designing new vaccines for controlling the disease. *Theileria* infected bovine lymphocyte cell lines were utilized for understanding the parasite diversity in India using microsatellite markers, *tams1* gene-based antigenic diversity and GBS analysis. The vaccine strain from India and the already published data for other worldwide *T. annulata* strains were used for understanding and comparing the diversity among the parasite cell lines.

The microsatellite data analysis showed high population diversity among Indian parasites, which is in line with previous findings from Oman, Tunisia, and Turkey though less diversity was reported from Portugal and China (Weir et al., 2007, 2011; Al-Hamidhi et al., 2015; Gomes et al., 2016; Yin et al., 2018). The presence of multiple genotypes in each sample represented by high MOI values has been previously shown to be linked to high transmission intensity in related parasites like *P. falciparum* (Pinkevych et al., 2015). The MOI values were found to be higher when compared with values reported from other countries (Weir et al., 2007, 2011; Al-Hamidhi et al., 2015; Gomes et al., 2016; Yin et al., 2018). The allelic analysis showed the presence of unique alleles when compared with previously reported alleles. The Indian parasite population was found to be in LE in contrast to LD that is reported for the majority of countries. In China, LE was observed region wise, however, LD was reported when the same analysis was done by taking samples from different regions as a population (Yin et al., 2018). The small sample size can be one of the reasons for the detection of LE in the Indian population and further studies with more samples are needed to verify the same. Despite the high genetic diversity, the population structure was found to be panmictic as represented using the PCoA analysis. The PCoA analysis clustered Indian parasites with Oman, Sudan, and Tunisia while least similarity was found with Turkey isolate. The Indian parasite population didn’t show any geographical sub-structuring based on the PCoA analysis when analyzed with other country strains like Oman, Tunisia, Sudan, and Turkey.

The *tams1* gene has been shown to be a promising candidate for carrying antigenic diversity studies in *T. annulata* parasites (Gubbels et al., 2000b; Wang et al., 2014). There are contradictory reports with respect to *tams1* gene sequence with some suggesting no geographic specificity and other showing region specificity based on the gene polymorphism (Gubbels et al., 2000b; Wang et al., 2014). Our results showed Indian parasite strains to be clustered in both group 1 and 2 which is different from the previous report which showed them to be present in only group 1 (Wang et al., 2014).

The GBS analysis also confirmed the genetic diversity among the Indian parasites. The analysis showed few strains from India to be similar to Ankara strain from Turkey based on the diversity in the parasite genome. The phylogenetic tree showed a panmictic parasite population in India based on the genetic diversity. The major limitation associated with next-generation sequencing methods like GBS is the purity of the DNA sample, which in case of *T. annulata* parasites is tricky as it is difficult to separate the pure parasite from the host lymphocyte cell. The presence of host DNA in the sample has led to low SNP call rate for the parasite in our GBS study. In future, we plan to use more powerful techniques like WGS with high coverage to better understand the genetic diversity and the genome of the Indian parasite strains.

The current study not only highlights the genetic and allelic diversity present among the Indian *T. annulata* but also compares it with the isolates reported from other countries. It also sheds light on the genetic and allelic diversity among the Indian parasite and the vaccine by the use of microsatellite marker, *tams1* sequencing and GBS. These findings indicate that a heterogeneous parasitic population is prevailing in India causing theileriosis and may render the vaccine ineffective due to high diversity. It is essential to perform genetic diversity studies including the parasite population from different parts of India to map the diversity among the overall natural parasite population. It will help in identifying new vaccine targets and policy to control the disease.

## Author Contributions

PS designed the study. SR, VB, DD, and SM performed the experiments. SR, VB, and PS compiled and analyzed the data. VB and PS wrote the manuscript.

## Conflict of Interest Statement

The authors declare that the research was conducted in the absence of any commercial or financial relationships that could be construed as a potential conflict of interest.
